# Morphological characterization of nevi on the caruncle conjunctiva under *in vivo* confocal microscopy

**DOI:** 10.3389/fmed.2023.1166985

**Published:** 2023-05-04

**Authors:** Jianhao Cai, Cangeng Xu, Tsz Kin Ng, Zeyi Li

**Affiliations:** ^1^Joint Shantou International Eye Centre of Shantou University and The Chinese University of Hong Kong, Shantou, Guangdong, China; ^2^Shantou University Medical College, Shantou, Guangdong, China; ^3^Department of Ophthalmology and Visual Sciences, The Chinese University of Hong Kong, Hong Kong, Hong Kong SAR, China

**Keywords:** pigmented nevus, lacrimal caruncle, conjunctiva, *in vivo* confocal microscopy, histopathology

## Abstract

**Objective:**

This study aimed to investigate the microscopic structure and characteristics of nevi on the conjunctiva of the lacrimal caruncle by *in vivo* confocal microscopy.

**Methods:**

In total, four patients with nevi growing on the lacrimal caruncle conjunctiva were recruited. The morphological characteristics of the nevi were evaluated by *in vivo* confocal microscopy before excision surgery; the results were compared with histopathological analyses of the surgical specimens.

**Results:**

The nevi of the four patients were all located at the conjunctiva of the lacrimal caruncle, with a slightly nodular surface, mixed black and brown color, and clear boundary. The nevi were round and highly protruded on the surface of the lacrimal caruncle, with an average diameter of 4.5 ± 1.29 mm. Under *in vivo* confocal microscopy, the pigmented nevus cells on the conjunctiva of the lacrimal caruncle were observed to be clustered in nests with irregular boundaries. The cells were round or irregular, with clear cell boundaries, hyper-reflective at the periphery, with low reflectivity in the center. Vascular crawling was observed in some regions. Histopathological analysis showed that nevus cells were roughly equal in size and distributed in a nodular pattern. Melanin granules were observed in the cytoplasm. No atypia or mitotic figures of the cells were found.

**Conclusion:**

This study revealed that the microstructure of nevi growing on the conjunctiva of the lacrimal caruncle can be identified by *in vivo* confocal microscopy.

## 1. Introduction

Conjunctival melanocytic lesions are the most common tumors of the conjunctiva, accounting for 52% of all conjunctival tumors. Among all conjunctival melanocytic lesions, conjunctival nevi are the most common, accounting for 45% (1). A conjunctival nevus is a freckle or mole-like spot on the conjunctiva. Clinical diagnosis of conjunctival nevus can be challenging under some conditions. Histopathological examination is still the gold standard for diagnosis of melanocytic lesions, but this requires surgical excision. A potential diagnostic tool to allow for earlier diagnosis, before surgical operation, could be *in vivo* confocal microscopy (IVCM), which is a real-time, non-invasive form of high-resolution microscopy. At present, IVCM imaging is mainly used for corneal diseases, and it has also been applied in the study of neoplasms of the ocular surface (2, 3). Morphological changes in lesions at the cellular level can be observed via IVCM, but only a small number of studies have presented IVCM images of nevi in the caruncle conjunctiva (4, 5). Here, we present a series of lacrimal caruncle conjunctival nevi investigated via IVCM before surgery, with these investigations compared with the histopathology analysis.

## 2. Methods

### 2.1. Study subjects

This study was approved by the Human Medical Ethics Committee of the Joint Shantou International Eye Center of Shantou University and the Chinese University of Hong Kong (approval number: EC20171103(6)-P01). Written informed consent was obtained from all study subjects after explanation of the nature and possible consequences of the study. In total, four patients (one male subject and three female subjects) were recruited between August 2018 and November 2021; all were undergoing surgical removal of a lacrimal caruncle conjunctival pigmented nevus. The exclusion criteria were: (1) the patient did not agree to undergo Heidelberg Retinal Tomography (HRT3) examination; and (2) acute inflammation on the ocular surface.

### 2.2. *In vivo* confocal microscopy examination

IVCM examination was carried out using a HRT3/Rostock Cornea Module (RCM; Heidelberg Engineering, Germany) equipped with a 670-nm laser (Supplementary Figure 1). The resolution of the IVCM output image was 384 pixels × 384 pixels, with 800 × magnification. Each image corresponded to a horizontal section of 400 μm × 400 μm, with a high resolution of up to 1 μm.

Before IVCM examination, topical anesthesia was applied to the inferior conjunctival fornix of the eye with 0.5% proparacaine eye drops, administered three times at 5-min intervals. Carbomer eye gel was applied to the tip of the IVCM, which was then covered with a disposable sterile transparent shade. In order to display the detected image more clearly, the outer layer of the contact sleeve was evenly coated with carbomer eye gel. After sufficient ocular surface anesthesia, the chin and forehead of the subject were fixed in the corresponding positions of the IVCM machine. The ophthalmologist (C.X.) extended the upper and lower eyelids of the examined eye with the middle finger and thumb, respectively. The IVCM lens was then advanced by the other hand of the ophthalmologist so that it touched the edge of the pigmented nevus under examination. The scanning depth was adjusted, and the scan was performed gradually, moving from shallow to deep. Horizontal scanning was performed from the temporal side of lesion to the nasal side, and IVCM images were captured at any time during the scanning process. The five clearest images from each patient were selected for analysis.

### 2.3. Excision surgery

All operations were carried out by a single surgeon (J.C.). Before surgery, topical anesthesia was applied to the inferior conjunctival fornix of the eye with 0.5% proparacaine eye drops, administered three times at 5-min intervals. Local anesthetic was administered to the caruncle subconjunctival tissue by injection with 0.5 ml of 2% lidocaine. The conjunctival tissue was incised by 1 mm beyond the edge of the nevus. The nevus and a small amount of subconjunctival tissue were completely removed and sent for histopathological examination. The conjunctival incision of the lacrimal caruncle was released and closed intermittently with 10-0 nylon suture. Attention was paid to avoid injury of the lacrimal canaliculus during the operation.

### 2.4. Histopathological examination

Histopathological examination was performed for all four specimens. The tissues of the caruncle conjunctival nevus were fixed in 10% paraformaldehyde, dehydrated, and embedded in paraffin according to the conventional procedure. The tissue sections (5 μm) were sliced, spread, baked, and stained with hematoxylin and eosin. Briefly, after deparaffinization at 60°C, the sections were incubated twice in xylene, then re-hydrated by passing them through decreasing concentrations of alcohol baths and water. The sections were stained for 8 min with Harris hematoxylin staining solution, followed by incubation with 1% hydrochloric acid in ethanol for 10 s. After washing, the blue-promoting solution appeared blue after 10 s. The sections were then washed with tap water, stained with 1% eosin iron-red, dehydrated in graded alcohol solutions increasing in concentration, and cleared in xylene. The HE-stained sections were imaged and analyzed via light microscopy by a pathologist to determine the morphological changes in the caruncle conjunctival nevus.

## 3. Results

### 3.1. Clinical diagnosis

**Figure 1:**
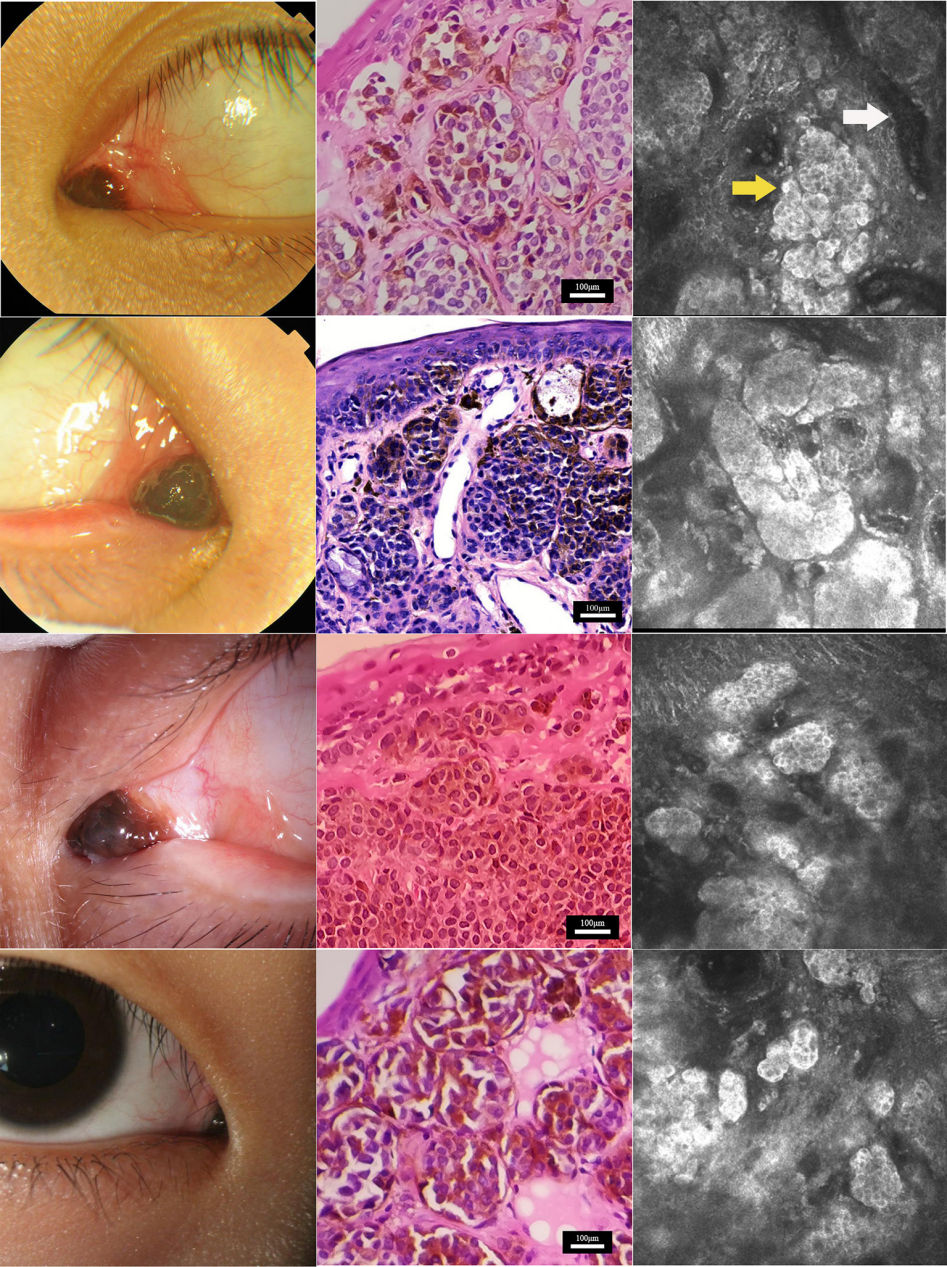
Characterization of nevus on the caruncle conjunctiva by histopathological and *in vivo* confocal microscopy analyses. Clinical **(left)**, histopathological **(center)**, and *in vivo* confocal microscopy **(right)** analyses of nevi at the caruncle conjunctiva in the four study subjects. Confocal microscopy showed that nevus cells (yellow arrow) in the conjunctiva of the lacrimal mound were clustered in nests with clear boundaries. The reflectivity of the nest was significantly increased compared with normal lacrimal mound tissue. Each single nevus cell was round or irregular, with clear cell boundaries. The periphery of the cell was hyper-reflective, and the center was hypo-reflective. Blood vessels (white arrow) were seen around the nevus cell nest in some areas. Scale bar for histopathological analysis: 100 μm. Depths of the *in vivo* confocal microscopy images captured: row 1, 14 μm; row 2, 50 μm; row 3, 23 μm; row 4, 25 μm.

Four patients, one male subject and three female subjects, were recruited. The ages of the four enrolled patients ranged from 14 to 24 years (mean: 17.75 ± 2.08). All lesions occurred only in one eye, with two occurring in the right eye and two in the left. The nevi of the four patients were all located at the conjunctiva of the lacrimal caruncle, with a slightly nodular surface, mixed black and brown color, and clear boundary (Figure 1). The nevi were round and highly protruded on the surface of the lacrimal caruncle, with an average diameter of 4.5 ± 1.29 mm (range: 3–6 mm).

### 3.2. Histopathological examination

The four surgical specimens from the patients were all diagnosed as pigmented nevus in the histopathology analysis. Microscopically, the pigmented nevi were completely removed, and no nevus cells were seen at the edges of the specimens. Hematoxylin and eosin staining showed that nevus cells were located within the epidermis and dermis (Figure 1). The nevus cells were roughly equal in size and distributed in a nodular pattern. Melanin granules were observed in the cytoplasm. No atypia or mitotic figures of the cells were found in any of the four samples.

### 3.3. *In vivo* confocal microscopy examination

**Figure 2:**
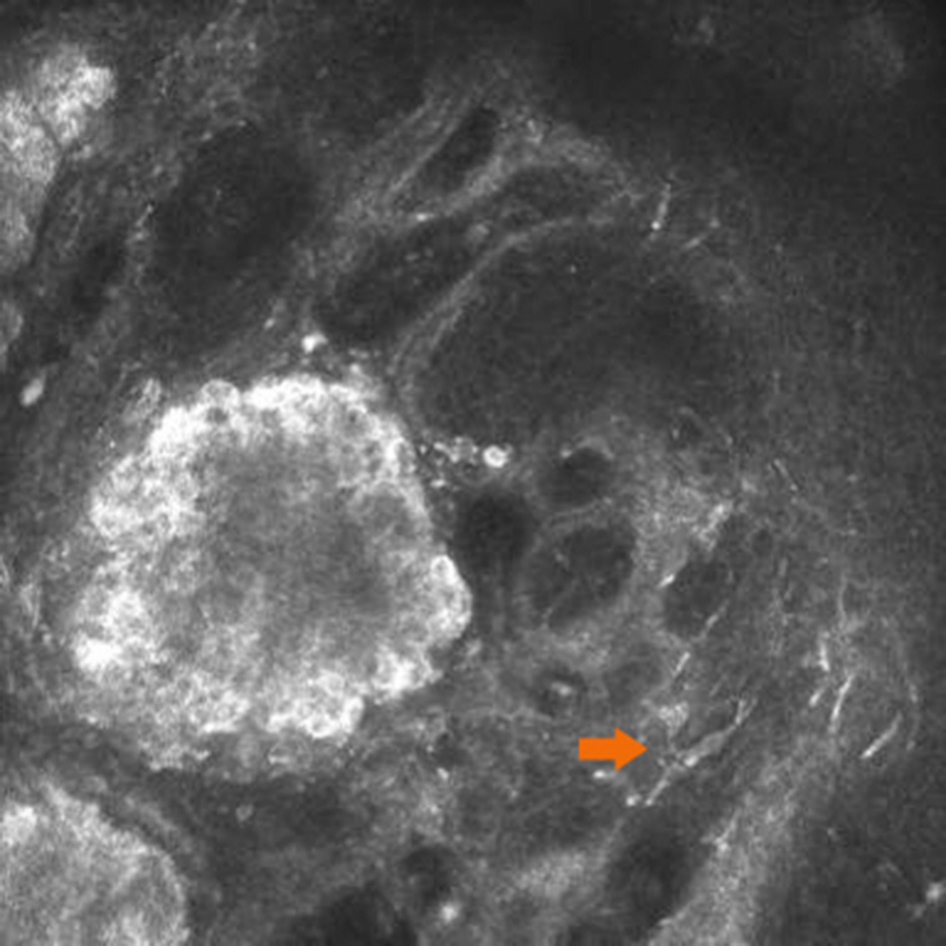
Langerhans cells around the lesions. Thick Langerhans cells (orange arrow) with irregular morphology were observed around the lesions in one of the four cases.

Under IVCM, nevus cells on the conjunctiva of the lacrimal caruncle were observed to be clustered in nests with clear and irregular boundaries, and reflectivity was significantly increased as compared to that of normal lacrimal caruncle tissue (Figure 1). Each single nevus cell was round or irregular, with clear cell boundaries, hyper-reflective at the periphery, with low reflectivity in the center. Blood capillaries were observed around the nevus cell nest in some areas. Thick Langerhans cells with irregular morphology were observed around the lesions in one of the four cases (Figure 2). The structure of the lacrimal caruncle conjunctival nevus observed under IVCM was similar in appearance to that of the histopathological section.

## 4. Discussion

A pigmented nevus, also known as a melanocyte nevus, is a benign hyperplastic growth composed of nevus cells. Its distribution covers the skin of the entire body, including the face and eyelid skin, as well as the conjunctiva. A Korean study has reported that 76.5% of conjunctival pigmented lesions are located on the bulbar conjunctiva, 8.2% on the caruncle, and 1.2% on the fornix. Compound nevus is the most common type (67.1%) of conjunctival melanocytic tumor and has a benign course. In contrast, conjunctival malignant melanoma is rare (7.1%) and carries serious consequences (6). Clinical diagnosis mainly depends on the history of the morphological evolution of the skin lesion, and suspicious cases require histopathological examination. Histopathological examination is still the gold standard for melanocytic lesions; however, it is invasive and cannot produce results in real time. New imaging techniques are of strong interest in the diagnosis of conjunctival pigmented neoplasms. IVCM is a non-invasive high-resolution imaging technique that has been demonstrated to be useful for the diagnosis of skin and ocular surface diseases (7).

There are two main kinds of IVCM device used in clinical settings. One is dedicated to the skin, with a handheld probe, and the other is applied to the ocular surface using the Rostock Cornea Module. Under IVCM, cells or tissues can have a similar appearance to the non-invasive optical slices visualized under CT scan. CT has been applied in dermatology for a significant period of time, and it can effectively identify the properties of lesions (8). However, the type of confocal microscope used for the skin cannot be used for the conjunctiva, especially the lacrimal caruncle, as there is a physiological depression in the lacrimal caruncle. It is difficult to achieve sufficient proximity to the larger probe, and lacrimal caruncle lesions cannot be detected (9).

The form of IVCM used in this study is a real-time, non-invasive, and high-resolution imaging method, providing magnification by up to 800 times, which can detect fine structures at the cellular level. In 1990, Cavanagh *et al*. reported the application of IVCM to observe and record human corneal stratification *in vivo* for the first time (10). Since then, with the continuous progress of science and technology, the research scope of IVCM has gradually expanded from initial application in the diagnosis of and research on corneal diseases to applications in other ocular surface diseases (11). At present, IVCM is mainly used in the examination and diagnosis of corneal diseases, meibomian gland dysfunction, and other related issues. Compared to skin CT, the IVCM probe used for ocular surface analysis is smaller, which makes it more suitable for examination of ocular surface diseases.

In this study, we found that IVCM images of lacrimal caruncle conjunctival pigmented nevi were highly similar to images of histopathological sections, demonstrating that IVCM can accurately visualize the microstructure of a lacrimal caruncle conjunctival pigmented nevus. We summarize the features of the IVCM images from the four patients as follows: (1) the boundaries of the lesions were clear, showing a nest distribution; and (2) dense, large, bright cells arranged in a honeycomb-like pattern with clear cell boundaries were observed within the lesions. The cell sizes were roughly uniform. Our findings were consistent with previously reported IVCM images of dermal melanocytic nevi (12). However, they differed from IVCM images of conjunctival nevi, which have been found to exhibit lower reflectivity than the surrounding tissue (9). This might be due to the fact that the tissue at the lacrimal caruncle is closer to the skin tissue. Pigmented nevi at this site are also relatively more protruded and more similar to intradermal nevi of the skin. Not only does IVCM display the structure of the lesion at the cellular level, but it also produces these images in real time. During the examination, the probe angle and scanning depth can be adjusted at any time to evaluate the lesion in different dimensions in order to obtain more information.

There were several limitations to this study. First, a limited number of study subjects were included. Second, IVCM has limited penetration and is easily affected by the light transmittance of the tissue. For superficial and transparent tissues, the imaging is clear, but for deep tissues or tissues with poor light transmittance, the imaging is fuzzy. Third, the resolution of IVCM is not comparable to that of conventional histopathological sections, although IVCM is non-invasive, real-time, and dynamic as compared to traditional histopathology, which is time-consuming and invasive. In addition, the examination process for IVCM requires the cooperation of the patient (12). Finally, IVCM is not able to distinguish activated Langerhans cells from dendritic melanocytes, since both are cells with prominent dendritic processes and they share similar refractive indices and morphological features. However, their distributions differ, in that Langerhans cells are more superficial and more common in the conjunctival tissue. The processes of Langerhans cells are also stouter than those of melanocytes.

## 5. Conclusion

This study revealed the ability of IVCM to display the microscopic structure of nevi of the lacrimal caruncle conjunctiva with a certain degree of recognition. Although the resolution of IVCM is not comparable to that of conventional histopathological sections, the non-invasive, *in situ*, real-time, and dynamic characteristics of IVCM enable it to overcome the disadvantages of traditional histopathological examination.

## Data availability statement

The original contributions presented in the study are included in the article/Supplementary material, further inquiries can be directed to the corresponding author.

## Ethics statement

The studies involving human participants were reviewed and approved by Human Medical Ethics Committee of Joint Shantou International Eye Center of Shantou University and The Chinese University of Hong Kong. The patients/participants provided their written informed consent to participate in this study. Written informed consent was obtained from the individual(s) for the publication of any potentially identifiable images or data included in this article.

## Author contributions

JC: conception and design, data collection and surgical operation, and drafting of the manuscript. CX: IVCM examination. JC, CX, and ZL: analysis and interpretation of data. TN: critical revision of the manuscript. All authors read and approved the final manuscript.
