# Supplementary material for: Morphological characterization of nevi on the caruncle conjunctiva under in vivo confocal microscopy
Source: Front Med (Lausanne). 2023 May 4;10:1166985. doi: 10.3389/fmed.2023.1166985 (PMC10194655; doi:10.3389/fmed.2023.1166985)
Supplement: Supplementary file 1 [file Data_Sheet_1.docx]

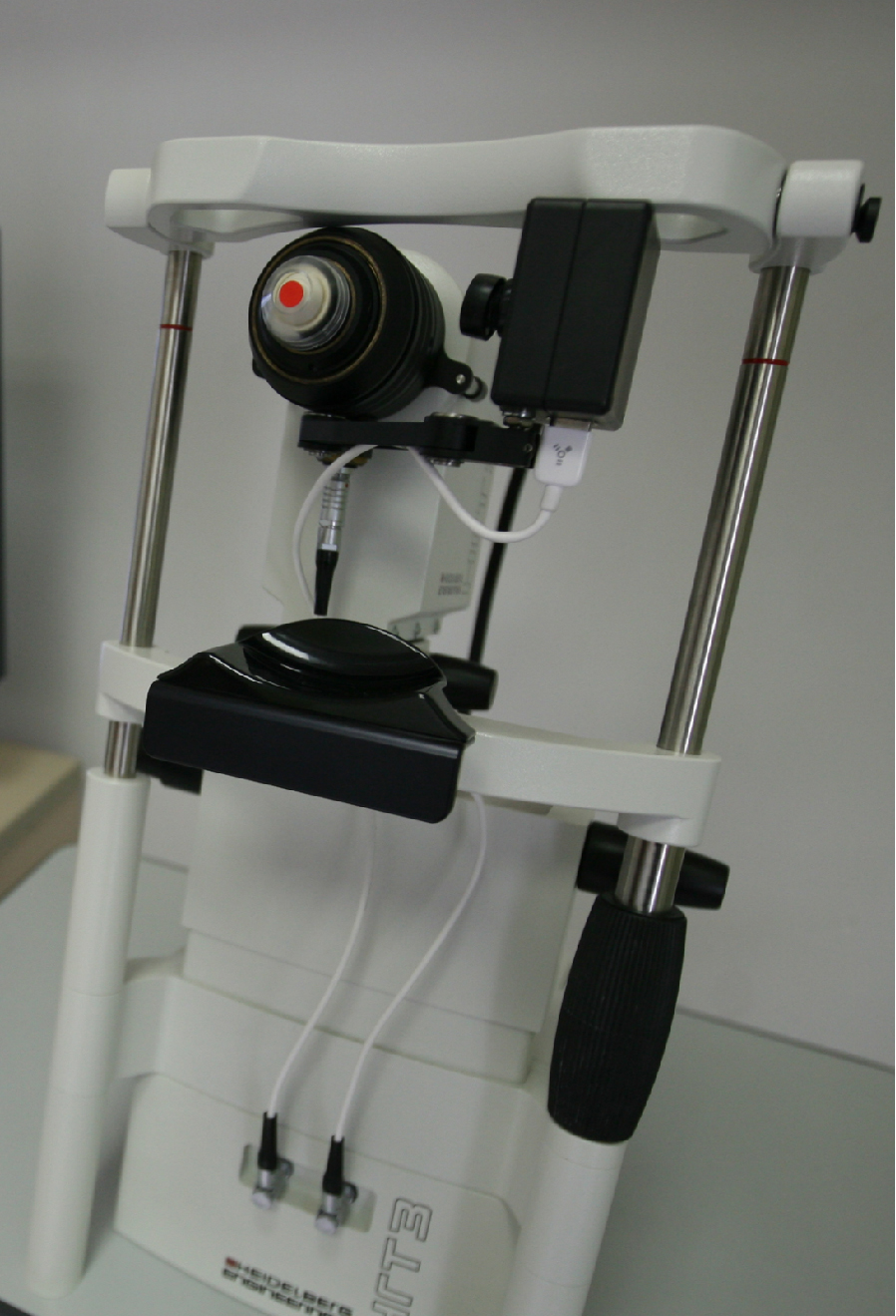


**Supplementary figure 1: The instrument for the *in vivo* confocal microscopy examination.**

The Heidelberg Retina Tomography 3/Rostock Cornea Module (Heidelberg Engineering, Germany).
